# Large serological survey showing cocirculation of Ebola and Marburg viruses in Gabonese bat populations, and a high seroprevalence of both viruses in *Rousettus aegyptiacus*

**DOI:** 10.1186/1471-2334-9-159

**Published:** 2009-09-28

**Authors:** Xavier Pourrut, Marc Souris, Jonathan S Towner, Pierre E Rollin, Stuart T Nichol, Jean-Paul Gonzalez, Eric Leroy

**Affiliations:** 1 Institut de Recherche pour le Développement, UR 178, Marseille, France; 2 Centre International de Recherches Médicales, BP 769, Franceville, Gabon; 3 Institut de Recherche pour le Développement, UR 178, Mahidol University at Salaya, 25/25 Phutthamonthon 4, Nakhonpathon 73170, Thaïland; 4 Special Pathogens Branch, Centers for Disease Control and Prevention, Atlanta, Georgia, USA

## Abstract

**Background:**

Ebola and Marburg viruses cause highly lethal hemorrhagic fevers in humans. Recently, bats of multiple species have been identified as possible natural hosts of *Zaire ebolavirus *(ZEBOV) in Gabon and Republic of Congo, and also of *marburgvirus *(MARV) in Gabon and Democratic Republic of Congo.

**Methods:**

We tested 2147 bats belonging to at least nine species sampled between 2003 and 2008 in three regions of Gabon and in the Ebola epidemic region of north Congo for IgG antibodies specific for ZEBOV and MARV.

**Results:**

Overall, IgG antibodies to ZEBOV and MARV were found in 4% and 1% of bats, respectively. ZEBOV-specific antibodies were found in six bat species (*Epomops franqueti, Hypsignathus monstrosus, Myonycteris torquata, Micropteropus pusillus, Mops condylurus *and *Rousettus aegyptiacus*), while MARV-specific antibodies were only found in *Rousettus aegyptiacus *and *Hypsignathus monstrosus*. The prevalence of MARV-specific IgG was significantly higher in *R. aegyptiacus *members captured inside caves than elsewhere. No significant difference in prevalence was found according to age or gender. A higher prevalence of ZEBOV-specific IgG was found in pregnant females than in non pregnant females.

**Conclusion:**

These findings confirm that ZEBOV and MARV co-circulate in Gabon, the only country where bats infected by each virus have been found. IgG antibodies to both viruses were detected only in *Rousettus aegyptiacus*, suggesting that this bat species may be involved in the natural cycle of both Marburg and Ebola viruses. The presence of MARV in Gabon indicates a potential risk for a first human outbreak. Disease surveillance should be enhanced in areas near caves.

## Background

*Filoviridae*, a family of single-stranded non segmented RNA viruses, comprises two genera, *marburgvirus *(MARV) and *ebolavirus *(EBOV), the latter comprising *Zaire ebolavirus *(ZEBOV), *Sudan ebolavirus *(SEBOV), *Cote d'Ivoire ebolavirus *(CIEBOV), *Reston ebolavirus *(REBOV) and *Bundibungyo ebolavirus *(BEBOV) [1,2]. Filoviruses cause hemorrhagic fever outbreaks in humans, with high case fatality rates (except for REBOV). Seventeen outbreaks and two isolated cases of human EBOV infection have been reported, in Democratic Republic of Congo (DRC) (1976, 1977, 1995, 2007, 2008), Sudan (1976, 1979, 2004), Gabon (1994, 1996, 1997, 2001), Republic of Congo (RC) (2001, 2003, 2005), Ivory Coast (1995) and Uganda (2000, 2007) [2-4]. Three outbreaks and four isolated cases of human MARV infection have been reported, in Germany and the former Yugoslavia (1967), Zimbabwe (1975), Kenya (1980, 1987), DRC (1998-1999), Angola (2005) and Uganda (2007) [5]. Consistent with this epidemiological pattern, ecologic niche modeling of outbreaks and isolated cases has suggested that Ebola hemorrhagic fever occurs in the rain forests of central and western Africa, and Marburg in more open areas of central and western Africa [6,7]. In Gabon and RC, most human ZEBOV outbreaks have occurred through contact with infected animal carcasses, especially great apes and duikers [8]. Bats were recently identified as potential reservoir species of these filoviruses [3,9,10]. During the ZEBOV outbreaks that occurred between 2001 and 2003 in Gabon and RC, numerous animals were captured in the search for reservoir species. Of the 1024 small mammals sampled, including 679 bats, ZEBOV-specific antibodies were detected in the serum of 16 (8%) of 192 fruit bats belonging to three species, namely *Hypsignathus monstrosus, Epomops franqueti*, and *Myonycteris torquata*; ZEBOV RNA was detected in pooled liver and spleen samples from 13 (5%) of 279 such bats [11]. Sequence analysis indicated that bats of these species might serve as a ZEBOV natural host. A large-scale serological survey of bats belonging to these three species was then conducted, with 1390 specimens captured between 2003 and 2006 in three regions of Gabon and in the epidemic border region with RC. The prevalence of ZEBOV-specific IgG was about 5% in bats from all four regions, and fell to about 1% at all four sites a year after the most recent Ebola outbreak, which occurred in 2005 in RC [12]. These results indicate that ZEBOV-specific antibodies are widely distributed in these bats in Gabon and RC, supporting their potential reservoir status. As a Marburg outbreak occurred in 2005 in Angola, 800 km from the Gabon border, we also tested bats for signs of MARV infection. Of 438 bats collected in Gabon and RC, MARV-specific IgG was detected in the serum of 29 of 242 specimens of *Rousettus aegyptiacus*, and MARV nucleotide sequences were detected in four members of 283 bats of the same species, suggesting that *R. aegyptiacus *bats may be a MARV reservoir [13].

In the present study, we documented the distribution of ZEBOV- and MARV-specific antibodies in bat communities living in forest areas of Gabon and northern RC. We report detailed serologic results for 2147 bats belonging to at least nine species sampled between 2003 and 2008.

## Methods

### Collection sites and periods

**Figure 1:**
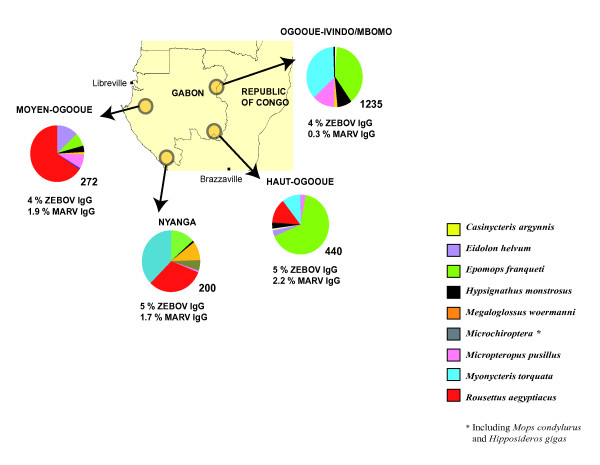
**Map of Gabon and Republic of Congo**. Sampling sites and sampled bat species are shown. The circles represent the sampling sites in Gabon and RC. The distribution (percentage) of the different captured bat species is represented by the different colors indicated in the box.

Samples were collected in the Republic of Congo and Gabon (Figure 1).

In the Republic of Congo, two sites were sampled near Mbomo (0°25' O; 14°41' E) and Odzala National Park. The vegetation includes dense semi-deciduous and evergreen forest with open and closed canopies. The climate is equatorial, with two dry seasons (December-February and June-August) and two wet seasons (March-May and September-November). In Gabon, bats were collected in three different regions. The first was situated in Haut-Ogooué province in the south-east of the country (near Franceville 1°37' S; 13°36' E). Four sites comprising forested areas, savannas and a cave were sampled. The vegetation forms a savanna-forest mosaic. The climate is tropical-transitional, with one clear dry season (June-August) and one long wet season (September-May), with lower rainfall in December and January. The second region was located in Moyen-Ogooué province in west Gabon (near Lambaréné 0°41' S; 11°01' E). Bats were collected from three different biotopes. The climate is tropical-transitional and humid. The forest vegetation is semi-deciduous with lakes, rivers and swamps. The third region was in Nyanga province, in south-west Gabon (near Tchibanga 2°51' S; 11°01' E). The vegetation is composed of an evergreen forest with savanna areas and caves. The climate is tropical with a long dry season (May-September) and a long wet season (October-April).

### Collection periods

In RC, bats were collected both during ZEBOV outbreaks in June 2003 and May 2005 and also one year after the last outbreak in May 2006. In Gabon, bats were collected in Haut-Ogooué in February 2005, October 2006 and March 2008, in Moyen Ogooué in April 2005, and in Nyanga in February 2006.

### Bat trapping and blood sampling

Bats were trapped with mist nets. The nets were set in the early evening, and captured animals were removed each morning. The captured bats were transported to a field laboratory specially designed for this study, a few kilometers from the capture sites, where they were euthanized. Weight and body measurements, sexing and species determination were performed as previously described [12]. Blood was collected in EDTA tubes by cardiac puncture and plasma was obtained by centrifugation at 3000 rpm for 10 min, then placed in Cryovials (VWR Prolabo, Fontenay-sous-Bois, France) and stored in liquid nitrogen. Fragments of liver and spleen were obtained by necropsy and immediately frozen. At the end of each capture period the samples were transported to Franceville for analysis at the Centre International de Recherches Médicales de Franceville (CIRMF), Gabon. For ethical aspects, a special authorization was delivered for this study by the Ministry of Health of Gabon (Pr Kombila Kouma Pierre, n°104/MSPP/SG/DGS, on 17 February 2005).

### Serological studies

Sera were tested with a standard IgG ELISA as previously described [12]. Briefly, ZEBOV antigens diluted 1:1000 in phosphate buffered saline were passively adsorbed overnight at +4°C onto ELISA Maxisorp plates (VWR Prolabo, Fontenay-sous-Bois, France). The protocol was the same for MARV antigens. Control wells were coated with uninfected cultured Vero cell antigens (mock antigens). The plates were washed three times with PBS-0.1% Tween 20. One hundred microliters of each serum sample diluted 1:100 in PBS-0.1% Tween 20 containing 5% fat-free milk powder (Becton Dickinson, Temse, Belgium) was added to each well and incubated overnight at +4°C. After three washes, 100 μl of a 1:1 mixture of peroxidase-labeled protein A (1 mg/ml) (Sigma, Taufkirchen, Germany) and peroxidase-labeled protein G (1 mg/ml) (Sigma, Taufkirchen, Germany) diluted 1:1000 in PBS-0.1% Tween 20 containing 5% fat-free milk was added to each well. The plates were incubated for one hour at room temperature and washed. TMB substrate (Thermo Electron Corporation, Saint Herblain, France) was used for detection. Optical density (OD) was measured at 450 nm with a PR 5100 ELISA plate reader (Biorad, Marnes-la-Coquette, France). All samples were also tested by using HPR-conjugated goat anti-bat IgG (1 mg/ml) (Bethyl Laboratories, Montgomery, USA) diluted 1:2000 in PBS-0.1% Tween 20 containing 5% fat-free milk. Samples from bats with corrected OD values > 0.31 (see below) were additionally tested at 1:400 and 1:1600 dilutions.

### Cut-off determination

**Figure 2:**
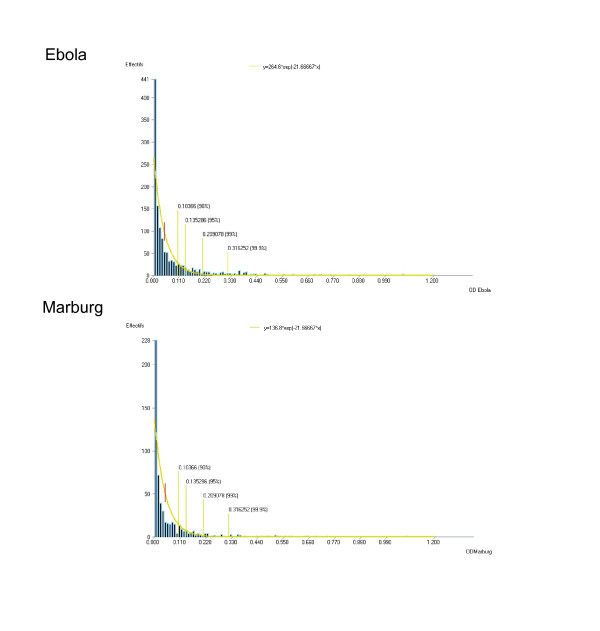
**Determination of ZEBOV- and MARV-specific IgG prevalence cutoffs**. The cut-off values were calculated assuming a negative exponential distribution of positive optical densities (OD). With a 0.001 risk of error, the cut-off was 0.31 for both Ebola and Marburg.

The specific activity of each serum at each dilution (corrected OD) was calculated by subtracting the non-specific background OD (wells with mock antigen) from the OD of wells with virus antigens. The cut-off value was calculated assuming a negative exponential distribution of positive OD values for negative bats. The parameters of the exponential distribution were derived from the distribution of the collected OD values and a risk of error in order to discard false-positives. With a 0.001 risk of error, the cut-off was 0.31 for both Ebola and Marburg viruses (Figure 2).

### Virological studies

Specific ZEBOV and MARV RNAs were detected in liver and spleen by means of real-time and nested PCR amplification as previously described [12,13]. For each bat, approximately 100 mg of tissue was incubated overnight at 4°C in 450 μl of cold 2× cellular lysis buffer (Applied Biosystems International, France) to inactivate viruses. Each tissue was then diluted to 1× and homogenized for 2 minutes, at 1500 strokes/min using a ball-mill tissue grinder (Geno/grinder 2000, BTC and OPS Diagnostics, Bridgewater, USA). Total RNA was extracted from ~150 μl of the homogenate and tested for ZEBOV or MARV using real-time or nested RT-PCR assays as previously described [12,13]. High-fidelity RT-PCR reagents (Biological Diagnostic Supplies Limited, Dreghorn, United Kingdom) were used for all conventional RT-PCR assays. ZEBOV nucleotide sequences [Genbank:DQ205409-DQ205415] and MARV nucleotide sequences [Genbank:EU068108-EU068113] of some bats tested in this study have previously been described [11,13].

### Statistical Analysis

Access software (Microsoft 2007) was used for data analysis. Seroprevalence rates were compared with the Chi-square test and Fisher's test, implemented with Epi Info software (6.04, Epiconcept, France). Statistical significance was assumed at p < 0.05.

## Results

### Bat collections

We collected 2147 bats belonging to at least nine species (Figure 1): 1235 specimens (58%) in Ogooué-Ivindo/Mbomo (8 species), 440 (20%) in Haut-Ogooué (8 species), 272 (13%) in Moyen Ogooué (7 species) and 200 (9%) in Nyanga (7 species). Most bats belonged to the species *Epomops franqueti *and *Myonycteris torquata *in Ogooué-Ivindo, *E. franqueti *in Haut-Ogooué, *Roussetus aegyptiacus *in Moyen-Ogooué and *R. aegyptiacus *and *M. torquata *in Nyanga. Bats of six species were present at all the sites, namely *E. franqueti *(8-64% of captured bats of this species by site), *Hypsignathus monstrosus *(1-8%), *Megaloglossus woermanni *(1-10%), *Micropteropus pusillus *(1-13%), *R. aegyptiacus *(1-66%) and *M. torquata *(1-36%) (Figure 1). Twenty-four members of the Microchiroptera were captured but they were not all identified to the species level, owing to a lack of expertise in the field and because voucher specimens were not preserved.

### Prevalence of ZEBOV- and MARV-specific IgG

#### Overall seroprevalence

**Figure 3:**
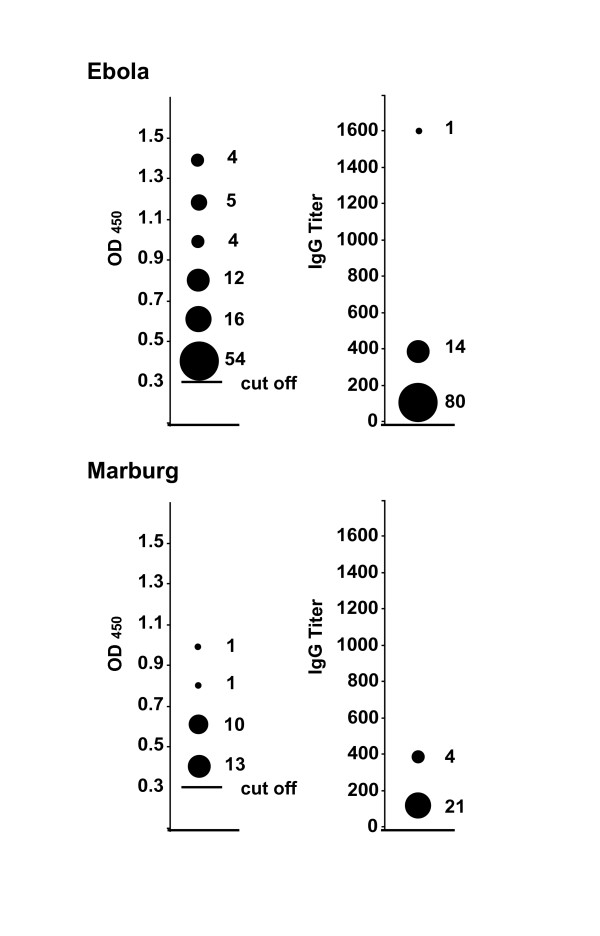
**Corrected ODs and antibody titers in ZEBOV - and MARV-positive bats**. The left side of the figure shows corrected OD values of 1:100-diluted bat sera with values higher than the 0.31 cutoff (solid horizontal bar). The numbers of sera used to calculate the values are shown to the right of the corresponding symbol. The right side of the figure shows IgG titers of sera with corrected OD values above 0.31. The numbers of sera used to calculate the values are shown to the right of the corresponding symbol.

Among the bats captured in Gabon and RC, 2147 were tested for ZEBOV-specific IgG and 1876 (87%) for MARV-specific IgG. The respective prevalence rates were 4% (n = 95) and 1% (n = 25) (Table 1). Sera from 15 of the 95 ZEBOV-positive bats had OD values > 0.31 when diluted 1:400, and one when diluted 1:1600 (Figure 3A). Sera from four of the 25 MARV-positive bats had OD values > 0.31 when diluted 1:400 (Figure 3B).

**Table 1:** *Marburgvirus *(MARV) and *Zaire ebolavirus *(ZEBOV) serological status (IgG) of bats captured between 2003 and 2008 in Gabon and Republic of Congo.

	**MARV**	**ZEBOV**
	
*Casinycteris argynnis*	0/18	0/18
*Eidolon helvum*	0/47	0/49
*Epomops franqueti*	2/679 (0.3)	36/805 (4)
*Hypsignathus monstrosus*	1/103 (1)	9/125 (7)
*Megaloglossus woermanni*	0/39	0/49
Microchiroptera*	0/20	3/24 (12)
*Micropteropus pusillus*	1/177 (0.6)	4/197 (2)
*Rousettus aegyptiacus*	21/299 (7)	24/307 (8)
*Myonycteris torquata*	0/493	19/573 (3)
Total	25/1876 (1)	95/2147 (4)

* including *Mops condylurus *and *Hipposideros gigas*

Data are numbers of IgG-positive bats versus numbers of captured bats, and percentages

#### Prevalence by species

ZEBOV-specific IgG was found in 4 (2%) of 197 *Micropteropus pusillus*, 19 (3%) of 573 *Myonycteris torquata*, 36 (4%) of 805 *Epomops franqueti*, 9 (7%) of 125 *Hypsignathus monstrosus*, 24 (8%) of 307 *Rousettus aegyptiacus *and 3 (12%) of 24 Microchiroptera (Table 1). The prevalence of ZEBOV-specific IgG was significantly higher (p = 0.005) in *R. aegyptiacus *specimens than in the bats of other species, with the exception of *H. monstrosus *(p = 0.82). The Microchiroptera group comprises several bat species and could not meaningfully compared with the bats of other species because of the small sample size. MARV-specific IgG was found in 21 (7%) of 299 *R. aegyptiacus*, 1 (1%) of 103 *H. monstrosus*, 1 (0.6%) of 177 *M. pusillus *and 2 (0.3%) of 679 *E. franqueti *(Table 1). Five *R. aegyptiacus *(3 from Haut-Ogooué, 1 each from Ogooué-Ivindo/RC and Moyen-Ogooué) were positive for both ZEBOV and MARV.

#### Prevalence by site and year

There were no significant differences (p = 0.7) in the prevalence of ZEBOV-specific IgG across the sites: 23 of 440 bats (5%) in Haut-Ogooué, 12 of 272 (4%) in Moyen Ogooué, 10 of 200 (5%) in Nyanga and 50 of 1235 (4%) in Ogooué-Ivindo/Mbomo (Figure 1). There was a temporal variation in the ZEBOV-specific IgG prevalence rates: in Mbomo (a region of documented Ebola outbreaks) the ZEBOV-specific IgG prevalence rate was 8 of 168 (5%) in 2003, 26 of 314 (8%) in 2005 and 10 of 494 (2%) in 2006. In Haut-Ogooué the ZEBOV-specific IgG prevalence rate was 10 of 154 (7%) in 2005, 3 of 130 (2%) in 2006 and 3 of 73 (4%) in 2008 (Table 2).

**Table 2:** *Zaire ebolavirus *(ZEBOV) serological status (IgG) of bats captured between 2003 and 2008 in Gabon and Republic of Congo, according to the collection site and period.

**Period of collection**	***E. franqueti***	***H. monstrosus***	***M. torquata***	**Total**
	
Mbomo 2003	4/100 (4)	2/18 (11)	2/50 (4)	8/168 (5)
Mbomo 2005	10/132 (8)	5/38 (13)	11/144 (8)	26/314 (8)
Mbomo 2006	5/221 (2)	2/39 (5)	3/234 (1)	10/494 (2)
Total	19/453 (4)	9/95 (9)	16/428 (4)	44/976 (4)
				
Haut-Ogooué 2005	8/109 (7)	0/1	2/44 (5)	10/154 (7)
Haut-Ogooué 2006	3/113 (3)	0/1	0/16	3/130 (2)
Haut-Ogooué 2008	3/59 (5)	0/12	0/2	3/73 (4)
Total	14/281 (5)	0/14	2/62 (3)	16/357 (4)

Data are numbers of ZEBOV IgG-positive bats versus numbers of captured bats, and (row) percentages. Only data for the three bat species suspected of being EBOV reservoirs are reported.

There were no significant differences (p = 0.9) in the MARV-specific IgG prevalence rates between Moyen-Ogooué (5 of 264, 1.9%), Haut-Ogooué (10 of 435, 2.2%) and Nyanga (3 of 180, 1.7%) but the MARV-specific IgG prevalence rates were higher (p = 0.003) in these regions than in Ogooué-Ivindo, where only three (0.3%) of 997 bats were positive (Figure 1).

Eighty-seven specimens of *R. aegyptiacus *were captured in caves (in Nyanga and Haut-Ogooué) and 220 were captured elsewhere (in Nyanga, Haut-Ogooué and Moyen Ogooué) (Table 3). MARV-specific IgG was detected in 12 (14%) of 84 cave-captured bats and in 9 (4%) of 215 bats captured elsewhere (p = 0.002). ZEBOV-specific IgG was detected in 8 (9%) of 87 *R. aegyptiacus *bats captured in caves and in 16 (7%) of 220 members of this species sampled elsewhere (p = 0.6) (Table 3).

**Table 3:** *Marburgvirus *(MARV) and *zaire ebolavirus *(ZEBOV) serological status (IgG) of bats of *Rousettus aegyptiacus *species in Gabon.

	**MARV**	**ZEBOV**
	
Cave	12/84 (14)	8/87 (9)
No cave	9/215 (4)	16/220 (7)
Total	21/299 (7)	24/307 (8)

Data are numbers of anti-ZEBOV IgG-positive bats versus numbers of captured bats, and (row) percentages.

#### Demographic parameters

No significant difference was found in the prevalence of ZEBOV-specific IgG between males (44 of 922, 4%) and females (51 of 1140, 4%) (p = 0.7) or between adults (79 of 1653, 5%) and juveniles (16 of 409, 4%) (p = 0.5) (Tables 4 and 5). However, the ZEBOV seroprevalence rate was higher (p = 0.004) in pregnant females (21 (8%) of 268) than in non-pregnant females (22 (3%) of 648). The prevalence of MARV-specific IgG was not different between males (13 of 822, 2%) and females (11 of 992, 1%) (p = 0.4), between adults (19 of 1444, 1%) and juveniles (5 of 373, 1%) (p = 0.9) or between pregnant females (3 of 209, 1.9%) and non pregnant females (4 of 577, 1%) (p = 0.3) (Tables 4 and 6).

**Table 4:** Demographic and ecological parametersassociated with *marburgvirus *(MARV) and *zaire ebolavirus *(ZEBOV) serological status (IgG) of bats captured between 2003 and 2008 in Gabon and Republic of Congo.

	**ZEBOV**	**MARV**
	
Male/female	No	No
Pregnant female/non pregnant	Yes	No
Adult/juvenile	No	No
Cave/elsewhere	No	Yes

'Yes' signifies statistical significance (p < 0.05) and 'No' no statistical significance. Seroprevalence rates were compared with the Chi-square test implemented in Epi Info software (6.04, Epiconcept, France).

**Table 5:** *Zaire ebolavirus *(ZEBOV) serological status (IgG) of bats captured between 2003 and 2008 in Gabon and Republic of Congo, according to demographic parameters.

	**Male**			**Female**					
				
	**Adult**	**Juvenile**	**Total**	**Adult Non pregnant**	**Adult pregnant**	**Juvenile**	**Total**	**ND**	**Total**
		
*C. argynnis*	0/4	0/0	0/4	0/9	0/4	0/1	0/14	0/0	0/18
*E. helvum*	0/11	0/7	0/18	0/17	0/0	0/11	0/28	0/3	0/49
*E. franqueti*	14/269(5)	2/87(2)	16/356(4)	9/243(4)	9/132(7)	2/36(6)	20/411(5)	0/8	36/805(4)
*H. monstrosus*	1/34(3)	2/19(11)	3/53(6)	1/18(6)	3/18(17)	2/33(6)	6/69(9)	0/3	9/125(7)
*M. woermanni*	0/19	0/0	0/19	0/21	0/5	0/4	0/30	0/0	0/49
*Microchiroptera*	1/9(11)	0/2	1/11(9)	2/5(40)	0/0	0/0	2/5(40)	0/8	3/24(12)
*M. pusillus*	0/76	0/3	0/79	1/36(3)	3/53(6)	0/22	4/111(4)	0/7	4/197(2)
*R. aegyptiacus*	11/88(13)	4/35(12)	15/123(12)	3/90(3)	3/13(23)	3/78(4)	9/181(5)	0/3	24/307(8)
*M. torquata*	9/227(4)	0/32	9/259 (3)	6/209(3)	3/43(7)	1/39(3)	10/291(3)	0/23	19/573(3)
Total	36/737(5)	8/185(4)	44/922(4)	22/648(3)	21/268(8)	8/224(4)	51/1140(4)	0/85	95/2147(4)

Data are numbers of ZEBOV-specific IgG-positive bats versus numbers of captured bats, and percentages.

**Table 6:** *Marburgvirus *(MARV) serological status (IgG) of bats captured between 2003 and 2008 in Gabon and Republic of Congo, according to demographic parameters.

	**Male**			**Female**					
				
	**Adult**	**Juvenile**	**Total**	**Adult Non pregnant**	**Adult pregnant**	**Juvenile**	**Total**	**ND**	**Total**
		
*C. argynnis*	0/4	0/0	0/4	0/9	0/4	0/1	0/14	0/0	0/18
*E. helvum*	0/11	0/7	0/18	0/16	0/0	0/11	0/27	0/2	0/47
*E. franqueti*	1/234(0.3)	0/80	1/314(0.3)	1/214(0.5)	0/88	0/31	1/333(0.3)	0/32	2/679(0.3)
*H. monstrosus*	0/32	0/15	0/47	0/14	0/13	1/28(4)	1/55(2)	0/1	1/103(1)
*M. woermanni*	0/16	0/0	0/16	0/15	0/5	0/3	0/23	0/0	0/39
*Microchiroptera*	0/8	0/0	0/8	0/5	0/0	0/0	0/5	0/7	0/20
*M. pusillus*	1/69(1)	0/3	1/72(1)	0/32	0/48	0/21	0/101	0/4	1/177(0.6)
*R. aegyptiacus*	10/87(11)	1/33(3)	11/120(1)	3/87(3)	4/13(30)	3/76(4)	10/176(4)	0/3	21/299(7)
*M. torquata*	0/197	0/26	0/233	0/185	0/38	0/35	0/258	0/15	0/494
Total	12/658(2)	1/164(0.6)	13/822(2)	4/577(1)	3/209(1)	4/206(2)	11/992(1)	0/62	25/1876(1)

Data are numbers of ZEBOV-specific IgG-positive bats versus numbers of captured bats, and percentages.

### Virus genetic analysis

ZEBOV nucleotide sequences were found in 13 (5%) of 279 bats sampled in 2003 in RC [11], and MARV nucleotide sequences were found in 4 (1%) of 283 bats sampled in 2005 in Gabon [13]. None of the other samples analyzed was PCR-positive, including those from bats with ZEBOV- or MARV-specific IgG.

## Discussion

The aim of this study was to elucidate the reported presence of ZEBOV- and MARV-specific antibodies in bats in Gabon. We had previously detected ZEBOV-specific IgG (in 16 of 192 bats) and ZEBOV RNA (in 13 of 279 bats) in *E. franqueti, H. monstrosus *and *M. torquata *specimens captured in the Ebola outbreak zone of northern Gabon and Congo between 2001 and 2003, and deduced that these three bat species might represent a ZEBOV reservoir [11]. Phylogenetic analyses of the seven sequences obtained from the 13 ZEBOV RNA positive bats showed very few differences relative to known human sequences (approximately 1% of nucleotide positions in the L gene), confirming that Ebola virus detected in bats corresponded to the ZEBOV species. We subsequently detected MARV-specific IgG (in 29 of 242 bats) and MARV RNA (in 4 of 283 bats) in specimens of *R. aegyptiacus *captured in southern and southwestern Gabon in 2005 and deduced that this species might represent a MARV reservoir. Phylogenetic analysis of the MARV sequences obtained from the bats, in the N and VP 35 genes, showed approximately 5% nucleotide divergence relative to the other western African Marburg virus lineage, Angola, a difference far smaller than the 15% diversity previously observed among East African Marburg virus isolates. This pointed to the existence of a geographic cluster including Angolan human MARV strains and Gabonese bat strains [13]. No blood samples were available from ZEBOV PCR-positive bats identified during our previous studies. However, all MARV PCR-positive bats were also positive for anti-MARV IgG [13].

Here we report the results of a large serological survey of ZEBOV and MARV in bats living in three regions of Gabon and northwest RC. In addition to the bats studied before, we examined additional specimens for ZEBOV infection (238 for IgG, 1468 for RNA) and for MARV (1438 for both IgG and RNA). We detected both ZEBOV-specific IgG (4%) and MARV-specific IgG (1%) in all four regions, confirming that the two viruses cocirculate in Gabon.

This is the first study to provide evidence of simultaneous ZEBOV and MARV circulation in bat populations in the same country. Several human ZEBOV outbreaks have occurred in Gabon, but no MARV outbreaks have been reported.

Interestingly, of the nine species reported here, specimens of six species (*E. franqueti, H. monstrosus, M. torquata, M. pusillus, R. aegyptiacus *and *Mops condylurus*) had ZEBOV-specific IgG and specimens of four species (*R. aegyptiacus, H. monstrosus, E. franqueti*, and *M. pusillus*) had MARV-specific IgG (Table 1). ZEBOV seroprevalence rates were high in four species, while a high MARV seroprevalence rate was found in only one species (*R. aegyptiacus*). Together with the elevated antibody titers (at least 1:400) found in these bats, these finding indicate that these bats have frequent contacts with ZEBOV and MARV.

In an earlier study in DRC, members of three bat species were found to be seropositive for MARV (*R. aegyptiacus *and two members of the Microchiroptera group: *Rhinolophus eloquens *and *Miniopterus inflatus*) [14], possibly because they roosted in close proximity at the capture sites. In Gabon, no specimens belonging to these last two species were captured during our field studies. Surprisingly, ZEBOV-specific IgG was also frequent in *R. aegyptiacus *(8%). No specimens of this species were sampled between 2001 and 2003 in northern Gabon/Congo [11,15]. ZEBOV seropositivity in *R. aegyptiacus *bats likely reflects true viral infection, although passive antigenic stimulation cannot be ruled out. In Gabon, Megachiroptera members, including *R. aegyptiacus, E. franqueti*, *M. torquata *and *H. monstrosus *bats, consume the same fruits (especially *Ficus *spp). Bats of different species tend to visit the same trees, and interspecies virus transmission (or simple antigenic stimulation) could occur via infected saliva deposited on fruits. Hendra virus and Nipah virus transmission has been suggested to occur in this way between bats belonging to Asian *Pteropus *species [16].

Among IgG-positive *R. aegyptiacus *specimens, 21 (42%) were positive for MARV, 24 (48%) were positive for ZEBOV, and 6 (10%) were positive for both viruses. Although dual infection and/or antigenic stimulation could explain the concomitant presence of ZEBOV- and MARV-specific IgG in individual bats, serological cross-reactions between Ebola and Marburg antigens cannot be ruled out.

The ZEBOV seroprevalence rates were very similar (4% to 5%) in the four sampling regions (Figure 1). By contrast, the MARV seroprevalence rate was higher (about 2%) in the three regions where *R. aegyptiacus *was abundantly captured. Although some groups of *R. aegyptiacus *may occasionally roost outside in the trees, the bats of this species usually roost in natural or man-made cavities (caves, mines, tombs, etc.) and search for food at night. The daily flight distances are unknown. Among the 299 *R. aegyptiacus *specimens sampled in Gabon, the MARV-specific IgG prevalence rate was significantly higher in the animals captured in caves (14%) than in those captured elsewhere (4%) (Table 3). This difference could be due to a higher circulation of MARV between the bats living in the caves. Similarly, a high MARV-specific IgG prevalence rate (about 20%) has been reported in DRC in a cave-captured population of *R. aegyptiacus *[14]. Together, these findings point to the existence of two different ecological biotopes for Marburg and Ebola viruses: one linked to bats roosting in caves (*Rousettus *or Microchiroptera species) and the other linked to bats roosting in trees in the open forest. The ecology of *H. monstrosus *depends on water supplies, and these bats are known to migrate locally, especially along rivers [17]. Thus, the geographic distribution of ZEBOV in its reservoir species may be very broad, encompassing the forested areas of Africa, especially close to rivers, as previously reported [6,7]. In contrast, the distribution of Marburg virus would be determined by the ecology (horizontal and vertical distribution) of *R. aegyptiacus *bats and the presence of caves. Our serological results strongly suggest that ZEBOV and MARV overlap in all four regions we sampled in Gabon and RC.

The prevalence of ZEBOV-specific IgG fell from about 5% in 2003 and 8% in 2005 in Mbomo and 7% in Haut-Ogooué (during ZEBOV outbreaks) to 2% at both sites in 2006 (one year after the last outbreak), before rising again to 4% in Haut-Ogooué in 2008 (Table 2). Similar seasonal variations in the IgG prevalence of other viral pathogens (European Bat Lyssavirus-1, Hendra virus, etc.) have also been described in bats [18,19]. These fluctuations could be due to factors such as changes in bat numbers (due to mortality, migration, influx of young animals, etc.), immunological parameters (antibody persistence) and environmental changes (fructification, etc.). Long-term studies of seasonal variations in the ZEBOV-specific IgG prevalence in bat populations might help to understand the dynamics of human Ebola outbreaks.

The higher prevalence of ZEBOV-specific IgG in pregnant females (8%) than in non pregnant females (3%) (Table 5) suggests that EBOV transmission between males and females could occur by contact with infected saliva (bites, licking) or by mating. Pregnant female *Pteropus scapulatus *bats in Australia were recently shown to be more susceptible than their non pregnant counterparts to Hendra virus infection, and it has been suggested that Hendra virus transmission may occur by contact with the infected placenta [19].

## Conclusion

We confirm the cocirculation of Ebola and Marburg viruses in Gabon. To date, Gabon is the only country where bat reservoirs of both viruses have also been identified. Of all the bats sampled in significant numbers in this survey, only specimens of *Rousettus aegyptiacus *were found to harbor both MARV- and ZEBOV-specific IgG, suggesting that this species may be a natural host of both viruses. The presence of MARV in Gabon, where no human Marburg hemorrhagic fever outbreaks have been reported, represents a potential and previously unrecognized threat to humans. As the MARV seroprevalence was significantly higher in *R. aegyptiacus *bats captured inside caves than elsewhere, caves and their surroundings should be considered as high-risk areas.

## Competing interests

The authors declare that they have no competing interests.

## Authors' contributions

Designed and performed the experiments: XP and EL. Analyzed the data: XP, EL, MS, PR, JT, SN and JPG. Statistical analysis MS. Contributed reagents/materials: PR, SN and JT. Wrote the paper: XP and EL. All authors read and approved the final version of the manuscript.

## Pre-publication history

The pre-publication history for this paper can be accessed here:

http://www.biomedcentral.com/1471-2334/9/159/prepub
